# *Schistosomiasis japonicum* diagnosed on liver biopsy in a patient with hepatitis B co-infection: a case report

**DOI:** 10.1186/1752-1947-8-45

**Published:** 2014-02-12

**Authors:** Victoria Parris, Kirsten Michie, Tim Andrews, Emmanuel F Nsutebu, S Bertel Squire, Alastair RO Miller, Mike BJ Beadsworth

**Affiliations:** 1 Tropical and Infectious Disease Unit, Royal Liverpool University Hospital, Prescot Street, Liverpool, Merseyside L7 8XP, UK; 2 Department of Pathology, Royal Liverpool University Hospital, Prescot Street, Liverpool, Merseyside L7 8XP UK; 3 Department of Clinical Sciences, Liverpool School of Tropical Medicine, Pembroke Place, Liverpool, Merseyside L3 5QA, UK

**Keywords:** China, Chronic hepatitis B virus, FibroScan®, Histology, Liver biopsy, *Schistosomiasis*, *Schistosomiasis japonicum*, Screening, Transient elastography, Viral hepatitis

## Abstract

**Introduction:**

Chronic hepatitis B virus and schistosomiasis are independently associated with significant mortality and morbidity worldwide. Despite much geographic overlap between these conditions and no reason why co-infection should not exist, we present what is, to the best of our knowledge, the first published report of a proven histological diagnosis of hepatic *Schistosomiasis japonicum* and chronic hepatitis B co-infection. A single case of hepatitis B and hepatic *Schistosomiasis mansoni* diagnosed by liver biopsy has previously been reported in the literature.

**Case presentation:**

A 38-year-old Chinese man with known chronic hepatitis B virus infection presented with malaise, nausea and headache. Blood tests revealed increased transaminases and serology in keeping with hepatitis B virus e-antigen seroconversion. A liver biopsy was performed because some investigations, particularly transient elastography, suggested cirrhosis. Two schistosome ova were seen on liver histology, identified as *S. japonicum*, probably acquired in China as a youth. His peripheral eosinophil count was normal, schistosomal serology and stool microscopy for ova, cysts and parasites were negative.

**Conclusion:**

Hepatic schistosomiasis co-infection should be considered in patients with hepatitis B virus infection who are from countries endemic for schistosomiasis. Screening for schistosomiasis using a peripheral eosinophil count, schistosomal serology and stool microscopy may be negative despite infection, therefore presumptive treatment could be considered. Transient elastography should not be used to assess liver fibrosis during acute flares of viral hepatitis because readings are falsely elevated. The impact of hepatic schistosomiasis on the sensitivity and specificity of transient elastography measurement for the assessment of hepatitis B is as yet unknown.

## Introduction

The geographical distributions of hepatitis B virus (HBV) and schistosomiasis overlap, and therefore co-infection may occur in patients. The ova of *S. mansoni* and *S. japonicum* reach the liver via the portal venous system and cause inflammation, leading to fibrosis and portal hypertension. It is possible to see schistosome ova on liver histology, but this has rarely been reported in the literature [1]. We present the case of a 38-year-old Chinese man with dual pathology of HBV e-antigen seroconversion causing acute liver inflammation and hepatic *S. japonicum* found incidentally on a liver biopsy.

## Case presentation

A 38-year-old Chinese man presented with a three-week history of anorexia, nausea and headache. He had been resident in the UK for 11 years and had been diagnosed with chronic HBV infection six months previously, at which time he was positive for hepatitis B surface antigen and e-antigen, and negative for e-antibody. His only other past medical history was of gastritis for which he had received *Helicobacter pylori* eradication therapy, and his only medication was lansoprazole. He occasionally drank alcohol and had a brother in China who also had chronic hepatitis B infection.

On examination, he had no stigmata of chronic liver disease and no hepatosplenomegaly. His admission blood test results demonstrated a rise in alanine aminotransferase (ALT) from 88U/L at diagnosis to 671U/L. His bilirubin level was 44μmol/L, alkaline phosphatase 125U/L, and gamma-glutamyl transpeptidase 125U/L. His level of albumin was normal at 38g/L. Clotting was mildly deranged (prothrombin time, 16.9 seconds; activated partial thromboplastin time, 45.6 seconds). A full blood count confirmed a longstanding thrombocytopenia (platelets, 69×10^9^/L) with a normal white blood cell differential.

His viral serology at the time of admission demonstrated that he had remained hepatitis B surface antigen positive, but was now e-antibody positive with persisting e-antigen, in keeping with e-antigen seroconversion. His hepatitis B viral load was high at log 8.3 (1.9×10^8^IU/mL). Serology also demonstrated previous hepatitis A, hepatitis E, Epstein-Barr virus and cytomegalovirus infections. Hepatitis C and hepatitis D serologies were negative, as were liver autoantibodies.

**Figure 1:**
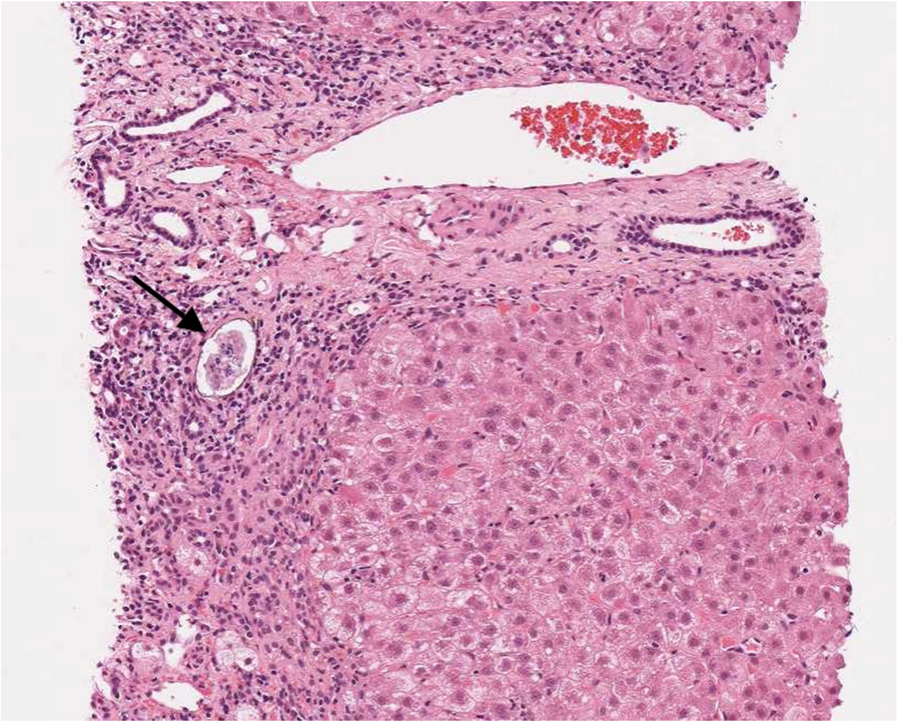
**Liver biopsy, scanned hematoxylin and eosin image × 10 magnification.** The schistosome ovum (arrowed) is situated next to a small bile duct and surrounded by inflammatory cells including lymphocytes and plasma cells. The inflammation involves a collapsed area of liver parenchyma; an adjacent nodule of hepatocytes is present. A portal tract in longitudinal section is present above the ovum.

**Figure 2:**
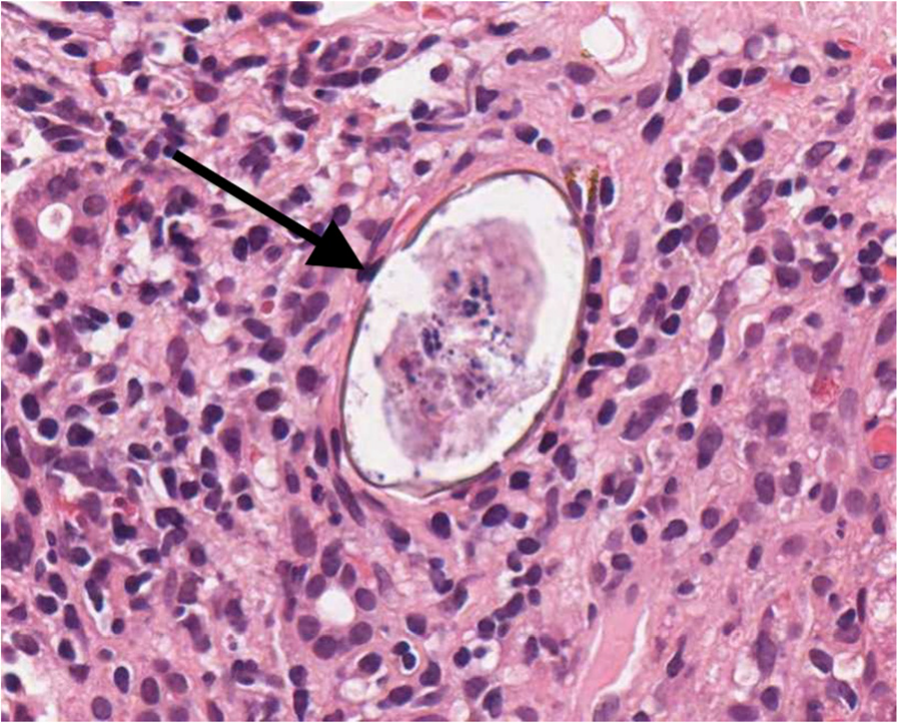
**Schistosome ovum within liver biopsy, scanned hematoxylin and eosin image × 40 magnification.** The schistosome ovum (arrowed) is non-operculated and thin-walled, measuring 79.2μm×48μm, morphologically consistent with *Schistosomiasis japonicum*.

An abdominal ultrasound scan demonstrated normal appearances of his liver and spleen with hepatopetal portal vein flow. Transient elastography (TE, FibroScan®) was performed (10 readings with 100% success, interquartile range/median value of 38.5%) with liver stiffness of 69.1kPa, consistent with liver cirrhosis (Metavir classification F4). Because of conflicting imaging results and biochemical markers in keeping with cirrhosis, we proceeded to an ultrasound-guided liver biopsy, the results of which were unexpected. The liver biopsy consisted of a 25mm core of tissue that included parts of 16 portal tracts. The architecture appeared nodular, which was due to parenchymal collapse as opposed to fibrosis, and this was accompanied by a patchy inflammatory cell infiltrate compromising lymphocytes, plasma cells and a small number of eosinophils. The appearance was of acute hepatitis but not specific with respect to its etiology. In addition, two ova were noted. These were non-operculated and thin-walled, measuring 79.2μm×48μm and 57.6μm×36μm, morphologically consistent with *S. japonicum* (Figures 1 and 2). There was no accompanying granulomata or pipestem fibrosis. Stool and urine microscopy did not identify any ova, cysts or parasites, and schistosomal serology was negative. His peripheral eosinophil count was normal (0.1×10^9^/L).

Our patient had grown up in Hubei province, China, and had not travelled abroad until moving to the UK 11 years ago. Since then he had returned to China on multiple occasions and had travelled within Europe. As an adolescent he had worked in paddy fields, a potential epidemiological exposure risk for *S. japonicum*, although this exposure was a long time in the past. Following the liver biopsy result, he was treated for schistosomiasis with 60mg/kg praziquantel in a single dose. At the time of treatment, his ALT level had significantly improved from a peak of 730U/L to 250U/L. Three months later, his ALT had normalized (30U/L) and hepatitis B serology continued to be in keeping with e-antigen seroconversion; his surface antigen remained positive, his viral load fell to log 4.8 (63,852IU/mL), and although his e-antibody was equivocal, he was only weakly positive for e-antigen.

## Discussion

Worldwide, over two billion people have been infected with viral hepatitis B, with an estimated 240 million people chronically infected, and 600,000 attributable deaths per year [2]. China is a highly endemic country although prevalence is decreasing with improved coverage of HBV vaccination of infants. Hepatitis B surface antigen prevalence decreased by 9.8% over 14 years to 7.2% in 2006, with a prevalence of 1% in children under five years [3]. However, variation occurs within China, particularly relating to ethnic group, education level and geography, with higher prevalence in the west compared to the east [3].

There are five species of schistosomes that can infect humans: *S. mansoni*, *S. haematobium*, *S. japonicum*, *S. mekongi* and *S. intercalatum*. In 2011, at least 243 million people required treatment for schistosomiasis, with transmission occurring in 78 countries [4]. *S. japonicum* is the only species infecting humans that is endemic to China, present in an area stretching from the China Sea to the Burmese border, along the course of the Yangzi river and upper Mekong delta [5]. At the peak prevalence of schistosomiasis in China during the 1950s, the highest prevalence of *S. japonicum* was found on alluvial deposits of the Yangzi river, including the Hubei province where our patient grew up [5]. Since the introduction of a control program in the 1950s, prevalence has decreased, but distribution of *S. japonicum* remains linked to agricultural areas using irrigation for rice cultivation, with seasonal peaks in transmission corresponding to spring floods and receding waters in autumn [5].

Few and conflicting data currently exist as to whether there is an interaction between HBV and schistosomiasis, with no current consensus [6]. Postulated reasons for the two infections co-existing include impaired cell-mediated immunity reducing host resistance; re-use of needles for parenteral treatment of schistosomiasis; and socioeconomic reasons, that is, those living in poverty have increased exposure to both viral hepatitis and schistosomiasis and so the two co-exist [6]. Studies suggest that patients with *S. mansoni* infection have a prolonged clearance rate of hepatitis B surface antigen, with Bassily *et al*. finding that patients with decompensated hepatosplenic schistosomiasis had a worse outcome if co-infected with HBV, with higher transaminases, more advanced changes on liver histology and higher mortality [7,8].

TE (FibroScan®) has been developed as a non-invasive, rapid, painless alternative to liver biopsy. TE measures liver stiffness, with values ranging from 2.5 to 75kPa. This value is then extrapolated to indicate the degree of liver fibrosis or cirrhosis. In our patient, the TE reading obtained was suggestive of liver cirrhosis, which was contrary to the findings on clinical examination, ultrasound of the liver and liver biopsy. TE has been validated for the diagnosis of significant fibrosis and cirrhosis in chronic hepatitis B and chronic hepatitis C, however it has limitations. Liver stiffness assessed on TE may be confounded when ALT is very high because necroinflammation causes falsely elevated readings of liver stiffness [9]. A study by Coco *et al.* of 10 patients with chronic viral hepatitis and acute exacerbations with ALT flares demonstrated a 1.3- to 3-fold increase in liver stiffness measurement [10]. Sagir *et al.* reported that liver stiffness readings using TE incorrectly suggested cirrhosis in 15 out of 20 patients with acute liver damage secondary to varying etiologies, including viral, drug-induced and autoimmune hepatitis [11]. In the case of our patient, the falsely elevated liver stiffness measurements would be in keeping with the ALT flare caused by hepatitis B seroconversion. We could find no published data on the effect of hepatic schistosomiasis on the validity of the use of TE for the assessment of liver fibrosis in viral hepatitis. It is possible that the development of Symmers pipestem fibrosis, the histological hallmark of hepatic schistosomiasis, could affect liver stiffness readings. This gap in knowledge is important as TE has advantages over liver biopsy in resource-poor settings endemic for schistosomiasis, such as portability and rapid operator training time, although the cost of equipment presents a barrier to its widespread use.

This case also raises the question as to whether immigrants and returning travellers should be screened for schistosomiasis. Although screening for schistosomiasis and other parasitic infections is appealing to treat and prevent their long-term sequelae, the best way to implement this is not clear. Studies have looked at the use of eosinophilia, schistosomal serology and stool microscopy for screening. The degree of peripheral eosinophilia in schistosomiasis relates to the stage, intensity and duration of infection, as well as the host reaction, limiting its use as an effective screening tool [12]. Bierman *et al.* found that a large number of returned travellers, expatriates and immigrants who were seropositive for schistosomiasis did not have an elevated eosinophil count, and the majority of those with eosinophilia were returned travellers with acute schistosomiasis, or immigrants with concomitant helminth infection [13]. Serological detection of schistosomiasis also has limitations: *S. mansoni* soluble egg antigen is commonly used for detection of *S. japonicum* infection due to greater availability than *S. japonicum* egg antigen, but although there is serological cross-reactivity between schistosomiasis species, sensitivity is reduced. Future serological diagnostics may be better - Smith *et al*. looked at the use of *S. mansoni* antigen derived from schistosomal cerceriae as a cheaper alternative to *S. mansoni* soluble egg antigen. They demonstrated that, for the diagnosis of *S. japonicum*, it performed as well as using *S. japonicum* egg antigen [14]. Epidemiological studies of schistosomiasis use stool and urine microscopy for the detection of infection. Increased numbers of Kato Katz smears performed increases schistosomiasis detection, although the optimum number of stool examined is debated. A study in Thailand reported that for all fecal parasites, microscopy of one stool identified 75% of parasites and two stools, over 90% [15]. Performing multiple stool microscopies is a labor-intensive process, with the potential for inter-operator variance, and therefore does not lend itself well as a screening tool. Hepatic *S. japonicum* infection in our patient was found incidentally on liver histology in the absence of peripheral eosinophilia, and with negative schistosomal serology and stool microscopy.

## Conclusion

Whether a relationship between HBV and hepatic schistosomiasis truly exists is not established, with few data on the impact of co-infection on disease progression, although hepatitis B surface antigen loss is less frequent in hepatic schistosomiasis co-infection [7].

In this report, we highlight the pitfalls of incorrectly using TE in the assessment of liver fibrosis during acute flares of viral hepatitis, when transaminases are grossly elevated and readings are falsely elevated. The impact of hepatic schistosomiasis on the sensitivity and specificity of TE measurement for the assessment of hepatitis B is as yet unknown, and an area for future work.

Hepatic schistosomiasis co-infection should be considered in patients infected with HBV from countries endemic for schistosomiasis. Perhaps, given the limitations of screening for schistosomiasis, and the uncertainty around the interaction between the two conditions, these patients should be treated presumptively if there are epidemiological reasons to suspect schistosomiasis.

## Consent

Written informed consent was obtained from the patient for publication of this case report and accompanying images. A copy of the written consent is available for review by the Editor-in-Chief of this journal.

## Abbreviations

ALT: Alanine aminotransferase; HBV: Hepatitis B virus; TE: Transient elastography.

## Competing interests

The authors declare that they have no competing interests.

## Authors’ contributions

VP and MB analyzed and interpreted the patient data regarding the hepatitis B and schistosomiasis co-infection. VP and KM were involved in the literature review and writing of the article. MB was involved in writing and editing the article. TA performed the histological examination of the liver and contributed to writing the article. SBS, EN and AM contributed to editing the article. All authors read and approved the final manuscript.
